# Hospital Length of Stay in Patients with and without Serious and Persistent Mental Illness: Evidence of Racial and Ethnic Differences

**DOI:** 10.3390/healthcare10061128

**Published:** 2022-06-17

**Authors:** Omolola E. Adepoju, Lyoung H. Kim, Steven M. Starks

**Affiliations:** 1 Department of Health Systems and Population Health Sciences, University of Houston College of Medicine, Houston, TX 77204, USA; sstarks@central.uh.edu; 2 Humana Integrated Health Systems Sciences Institute, University of Houston, Houston, TX 77204, USA; lkim2@central.uh.edu

**Keywords:** length of stay, inpatients hospital stay, racial/ethnic differences, serious and persistence mental illness, mental health equity

## Abstract

Background: Prior studies have documented racial and ethnic differences in mental healthcare utilization, and extensively in outpatient treatment and prescription medication usage for mental health disorders. However, limited studies have investigated racial and ethnic differences in length of inpatient stay (LOS) in patients with and without Serious and Persistent Mental Illness. Understanding racial and ethnic differences in LOS is necessary given that longer stays in hospital are associated with adverse health outcomes, which in turn contribute to health inequities. Objective: To examine racial and ethnic differences in length of stay among patients with and without serious and persistent mental illness (SPMI) and how these differences vary in two age cohorts: patients aged 18 to 64 and patients aged 65+. Methods: This study employed a retrospective cohort design to address the research objective, using the 2018 Healthcare Cost and Utilization Project (HCUP) National Inpatient Sample. After merging the 2018 National Inpatient Sample’s Core and Hospital files, Generalized Linear Model (GLM), adjusting for covariates, was applied to examine associations between race and ethnicity, and length of stay for patients with and without SPMI. Results: Overall, patients from racialized groups were likely to stay longer than White patients regardless of severe mental health status. Of all races and ethnicities examined, Asian patients had the most extended stays in both age cohorts: 8.69 days for patients with SPMI and 5.73 days for patients without SPMI in patients aged 18 to 64 years and 8.89 days for patients with SPMI and 6.05 days for patients without SPMI in the 65+ cohort. For individuals aged 18 to 64, differences in length of stay were significantly pronounced in Asian patients (1.6 days), Black patients (0.27 days), and Native American patients/patients from other races (0.76 days) if they had SPMI. For individuals aged 65 and older, Asian patients (1.09 days) and Native American patients/patients from other races (0.45 days) had longer inpatient stays if they had SPMI. Conclusion: Racial and ethnic differences in inpatient length of stay were most pronounced in Asian patients with and without SPMI. Further studies are needed to understand the mechanism(s) for these differences.

## 1. Introduction

Growing evidence continues to indicate the impact and burden of mental illnesses on U.S. adults. Mental illnesses are large contributors to the use of health-care services, disability and premature mortality [1]. In 2020, 52.9 million U.S. adults experienced any mental illness, and 14.2 million adults experienced serious mental illness, representing 21.0% and 5.6% of all U.S. adults, respectively [2]. Recent evidence suggests that, at any given time, more than 18% of adults in the United States suffer from any mental illness and over 4% suffer from a seriously debilitating mental illness [3,4].

Racialized groups tend to be less likely to seek mental health treatments [5,6,7], have less access to mental health services, and are less likely to receive appropriate mental health care [8]. These patterns in access to mental health care have persisted for years [9,10]. Among African Americans, the fear of encountering implicit bias and racism during care and long-standing trust issues in the US healthcare system contribute to lower mental healthcare utilization, which in turn is associated with overall poor health outcomes [4]. Additionally, mental illness stigma tends to be higher among racialized groups [5]. For example, among Asian Americans, cultural barriers, stigma toward mental health problems and treatments, and acculturation are significant contributors to lower mental health utilization rates [11,12].

When seeking and receiving care and treatment, patients with mental health disorders may experience longer lengths of stay (LOS). One study found that stays are longer at hospitals with a greater proportion of patients with serious mental illness and that the average length of inpatient stay for patients with serious mental illness is 10 days [13]. Others have identified factors related to longer LOS to include marital status [14], treatment procedures [15], as well as farther travel distance to the clinic [16]. Prior evidence suggests that racial and ethnic disparities exist in hospital LOS for mental health treatments as well. For instance, in the late 1990s and early 2000s, Black patients admitted to psychiatric inpatient care [17] experienced longer LOS than non-Black patients [18]. More recently, studies on the impact of the Affordable Care Act (ACA) on mental health utilization reported increased outpatient visits but no significant changes in mental health related hospitalizations, emergency department visits or prescription fill [19]. There is, however, a paucity of current literature on the recent relationship between race/ethnicity and mental health LOS in inpatient settings. Considering the impact of the COVID-19 pandemic on mental health [20], and the disproportionate COVID-19 morbidity and mortality burden experienced by racial and ethnic minorities [21], an assessment of racial and ethnic differences and length of inpatient stay (LOS) may contribute to our understanding of health inequities in mental health care.

Given the aforementioned factors, it is worthwhile to investigate racial and ethnic disparities in inpatient LOS for mental health treatments. While a 2014 study found no racial and ethnic differences in LOS between White and Asian American mental health patients [22], more recent studies of this nature are limited. This gap in research warrants further evaluation of the complex relationship among race, mental health, hospital inpatient utilization, and LOS, and can improve our understanding of the current trends in mental health equity across race and ethnicity. This study examined racial and ethnic differences in length of stay among patients with and without serious and persistent mental illness (SPMI). We examined two age cohorts (18–64 and 65+) to determine whether the observed differences in LOS vary because of older age and Medicare eligibility. Because mental health burden in older adults might vary from working adults’, this approach allows us to tease out differences between these cohorts.

## 2. Materials and Methods

### 2.1. Study Design

This study used a retrospective cohort design to examine racial and ethnic differences in LOS among patients with and without severe and persistent mental illness (SPMI), as well as how these differences vary in two age cohorts: patients aged 18 to 64 and patients aged 65+. We obtained data from the 2018 Healthcare Cost and Utilization Project (H-CUP) National Inpatient Sample (NIS) from the Agency for Healthcare Research and Quality (AHRQ). H-CUP is a reliable data source to understand estimates of health care utilization, cost, quality, and outcomes at the national level and to identify health care utilization by race and ethnicity, including state-level, hospital-level, and patient-level information [23]. The NIS contains information on all hospital stays and patient socioeconomic characteristics with discharge-level and hospital-level files [23]. This study merged the 2018 NIS Core file, which includes patient-level information such as age, sex, insurance type, number of diseases, and patient location, with the 2018 NIS Hospital file, which includes hospital information such as type and size of hospital.

The inclusion criteria were (1) patients with a diagnosis of mental illness in any diagnosis position and (2) patients aged 18+ diagnosed with a mental health disorder in 2018. Medical claims data were used to identify individuals with SPMI (using F-series ICD-10 codes), as defined by the Diagnostic and Statistical Manual of Mental Disorders, Fifth Edition (DSM-5). Patients who died during the study period were excluded from the analysis.

### 2.2. Measures

The primary outcome of this study was inpatient LOS, a continuous variable indicating how many days each patient stayed in the hospital in 2018. Race/ethnicity was the key independent variable comprising four categories as follows: (1) Non-Hispanic White, (2) Hispanic/Latino, (3) Non-Hispanic Black, (4) Non-Hispanic Asian/Pacific Islander, and (5) Non-Hispanic Native American/Other. Models examining associations between race and LOS among persons with mental illness were stratified by SPMI vs. non-SPMI, to understand how LOS varies with mental illness severity. We applied the definition of SPMI from the Milliman Integrated Report [24], which defines SPMI as having bipolar, major depression, or schizoaffective disorder [24]. To that end, patients with a diagnosis of bipolar (F31.XX), major depression (F33.XX), or schizophrenia disorder (F20-F29) were flagged as having SPMI, while other mental illness patients were flagged as non-SPMI.

This study adjusted two levels of characteristics as covariates to estimate length of inpatient stay. Patient-level variables included sex, age (18–24,25–34, 35–44, 45–54, 55–64, 65–74,75–84,85+), income quantile (0–25,26–50,51–75,76–100), the number of diagnoses (1–2, 3–5, 6+), specific comorbidities of interest as a binary variable (substance use disorder, alcohol disorder, heart failure, hypertension, chronic pain, diabetes), admission type as a binary variable (elective vs. non-elective), and patient’s geographic residence as a 5-category variable (Fringe counties of metro areas of >=1 million population; Counties in metro areas of 250,000–999,999 population; Counties in metro areas of 50,000–249,999 population; Micropolitan counties and non-metropolitan areas). Hospital-level characteristics, based on earlier studies, refs. [25,26] included type of hospital (private or teaching hospital), number of beds (small vs. medium and large), hospital ownership (invest-owned private vs. government owned hospital) and hospital geographic location (urban teaching, urban non-teaching, rural).

### 2.3. Statistical Analyses

Univariate analysis was used to describe patient and hospital characteristics by age cohort: 18–64 and 65+. The Kruskal Wallis test examined bivariate associations between mean LOS and sample characteristics. A multivariate generalized linear model (GLM) examined the relationship between race/ethnicity, SPMI status and LOS, with an interaction term between race and SPMI status. Considering LOS is a skewed continuous variable, GLM is appropriate because it gives a set of alternative functional forms to balance the relationship between the predicted value of the dependent variable and the linear index of the covariates. Predicted means for LOS were calculated to find precise differences between races and SPMI status. Sample weight was applied to the analyses according to HCUP’s sampling design with using the STATA 17 program. This project was ruled exempt by an independent institutional review board in December 2021. For confidentiality purposes, all elements that may directly identify a patient are removed from the HCUP datasets. HCUP is consistent with HIPAA regulations and, thus, HCUP databases are classified as de-identified data sets. Under HIPAA, review by an institutional review board (IRB) is not required for use of such data.

## 3. Results

Table 1 shows a summary of the key characteristics of the analytical sample. The sample comprised 1,523,859 patients between the ages of 18 and 64 and 1,053,093 patients older than 64 who were diagnosed with mental health disorders in 2018. The majority of the sample for both age groups was White: 66.4% of mental health patients between 18 and 64 were White while 78.4% of mental health patients older than 64 were White. Among patients between 18 and 64 years of age with mental health disorders, 18.7% were diagnosed with SPMI. Among older adult patients, 7.1% were diagnosed with SPMI.

Table 2 compares the unadjusted mean LOS by sample characteristic between the two age cohorts. On average, hospital stays were longer for patients from racialized groups compared to White patients. Asian patients had the longest average extended stay for both age groups. Patients aged 18 to 64 with SPMI had a longer average LOS (6.28 days) than those without SPMI. Similar patterns were observed for older adults: average LOS for patients with SPMI was 7.3 days, while average LOS for those without SPMI was 5.3 days. Patients aged 18 to 64 with SPMI had a shorter average LOS than those aged 65+ with SPMI across all race and ethnicity groups. For example, White patients aged 18 to 64 stayed an average of 6.22 days, and those aged 65+ stayed an average of 7.27 days.

Table 3 shows the weighted predicted mean for LOS for each racialized group for patients with and without SPMI and the changes in racial and ethnic differences in inpatient LOS for patients with and without SPMI. Patients from racialized groups had longer LOS than White patients, regardless of serious mental health status. Notably, Asian patients had the longest LOS irrespective of severity, both with and without SPMI, and the longest LOS in both age groups. The average LOS for patients aged 18 to 64 years was 5.73 days for non-SPMI patients and 8.69 days for SPMI patients. The average LOS for patients older than 64 years was 6.05 days for non-SPMI patients and 8.89 days for SPMI patients.

The magnitude of increase of the White-Asian difference in LOS was most considerable both for Asians aged between 18 and 64 and Asians aged 65+: 1.60 days (*p* < 0.01) and 1.09 days (*p* < 0.01), respectively. In contrast, White-Hispanic differences in LOS between non-SPMI and SPMI decreased significantly for older adults: 0.61 days fewer (*p* < 0.01). The White-Black difference in LOS significantly increased for Black people aged between 18 and 64: 0.27 days (*p* < 0.01).

## 4. Discussion

In this large national study examining racial and ethnic differences in LOS among patients with and without SPMI, we found evidence that racialized groups diagnosed with mental health disorders have, on average, longer LOS in hospitals than their White counterparts. Differences in LOS between White and racialized groups widens in patients with SPMI, when compared to patients with Non-SPMI diagnosis. After covariate adjustment, our findings reveal that for both age groups of study (18–64 and 65+), Asian patients exhibited the greatest disparity gap in LOS. 

Our finding of longer inpatient stays for racialized patients aligns with earlier studies that suggest they have longer inpatient stays for mental health treatment [18,22,27,28,29,30]. We posit that racialized patients tend to delay their treatments [31,32], and delay of care could lead to adverse mental health outcomes and higher illness severity at the time of admission [22,29,33], leading to longer stays in the hospital. In addition, this delay could be as a result of limited availability of culturally and linguistically appropriate mental health services or, conversely, that the use of interpretative services can prolong time to care. Already, more than 115 million individuals in the United States live in areas with shortages of mental health professionals [31] so that the availability of culturally and linguistically appropriate mental health services is further limited. Leveraging the healthcare system through the use of telehealth, for example, offers the potential to bridge the gap between mental health workforce shortage and increasing demand for mental health care.

Social and cultural determinants for delay in seeking health care can contribute to longer inpatients’ stay. For example, one study found that the stigma toward psychosis delays timely treatment; and an increase in the threshold for initiation of treatments manifests as longer untreated time and could eventually result in longer hospital stays [31]. Particularly for Asian subpopulations, earlier works suggest that cultural and/or familial factors can affect delay of the receipt of mental health treatments [34,35,36]. Social or familial networks may discourage access to health care services for patients with SPMI, as the family may not actively help family members with SPMI to seek healthcare services [32]. This is also true for treatment-seeking among Black Americans in that stigma toward mental health treatment reduces their likelihood of engaging in treatment [37]. Coupled with the cultural stigma in accessing care, limited trust in the healthcare system due to historic and present-day harms could contribute to delays that may further contribute to longer hospital stays [22,33,38,39]. Not surprisingly, these factors that explain disparities in LOS are also likely to associated with structural racism. For example, racialized groups are more likely to delay care due to cost-related reasons, longer travel distances to their healthcare providers [40,41] or mistrust of the mental healthcare system from experiences of racism [42]. An increase in mental health providers who have similar social and cultural backgrounds could positively influence racialized groups to access mental health treatments.

Diagnostic bias and treatment inadequacy for racialized groups can also explain the findings of this study. Previous research shows that Black and Hispanic patients have higher rates of misdiagnosis and overinterpretation of their mental health symptoms. These groups have also been found to have lower initiations and adequacy of care with fewer psychotropic drug fills than their White counterparts [38], which may lead to longer extended stays in hospital once they are admitted. Therefore, understanding the contributors to the delay in mental health services use and developing interventions for early initiation of mental health treatments has the propensity to improve mental health equity across race and ethnicity. Examining the quality of inpatients’ treatment by race and ethnicity can also inform future studies to improve our understanding of racial and ethnic differences in mental health LOS.

In comparing LOS between SPMI and non-SPMI patients, the findings of this study provide evidence that the differences in LOS between White and racialized groups become larger if patients have a SPMI diagnosis. For patients in the study aged 18 to 64, the magnitude of difference increased by 0.27 days for Black patients, 1.60 days for Asian patient, and 0.76 days for Native American patients/patients from other races. This aligns with findings from studies reporting that racialized groups with SPMI face greater disparities in seeking mental health services and treatment. These underrepresented groups are also less likely to receive outpatient care after hospitalization, have poorer mental health outcomes after discharge, are more likely to drop out of treatment, and are more likely to use emergency services than community support services when compared to non-Hispanic White patients with SPMI [37,41,42,43].

Over-representation of racialized groups in SPMI can also be a possible explanation of this finding. For example, Black Americans are more likely to be diagnosed with schizophrenia [18,39,43], which may extend their inpatient hospital LOS. Adequacy and quality of care during hospitalization may also account for LOS, and evidence suggests that race and ethnicity varies with SPMI [44]. Lower treatment quality for racialized groups [45] with SPMI may result in unfavorable outcomes of treatment, which could lead to longer stay in hospitals.

Finally, our findings suggest that racial and ethnic differences in inpatient LOS exist regardless of age group, which highlights race and ethnicity as critical factors in explaining mental health equity, rather than age. Therefore, behavioral health policies need to focus on social or cultural factors for disadvantaged populations in order to achieve mental health equity.

### Limitations

This study has several limitations. First, the study does not adjust for certain socioeconomic-related variables such as education and employment status because the H-CUP does not include those variables. Second, this study did not adjust patients’ perceived needs for mental health services, though some evidence suggests that this can contribute to mental health service use. Future studies should incorporate measurement of the quality of treatment for inpatient stays to better understand the association between LOS and race and ethnicity. Finally, the categorization of SPMI in this study only compared those with SPMI to those without SPMI; in reality, there could be a third subgroup of patients who have both SPMI and non-SPMI.

## 5. Conclusions

This study examined the association between length of inpatient stay and SPMI diagnosis across race and ethnicity groups. Overall, Asians, Blacks and Native Americans with mental health disorders stayed significantly longer than their White counterparts. Of all races and ethnicities examined, Asian patients had the longest stays for inpatient treatment in both 18–64 and 65+ age cohorts. Further, the differences in LOS between Whites and racial and ethnic minority groups increased significantly if patients had an SPMI diagnosis. Strategic initiatives to moderate racial and ethnic differences through interventions that incorporate both cultural sensitivity and financial assistance are warranted to improve mental health equity across all races and ethnicities.

## Figures and Tables

**Table 1:** Summary of Patient and Hospital Characteristics by Age Cohort: 18–64 and 65+.

		18–64	65+
		*n* = 1,523,859 *n* (%)	*n* = 1,053,093 *n* (%)
Race	Whites	1,014,738 (66.6)	826,370 (78.5)
	Black	282,044 (18.5)	109,198 (10.4)
	Hispanic	151,625 (9.9)	73,148 (6.9)
	Asian/Pacific Islander	20,418 (1.3)	18,110 (1.7)
	Native American/other	55,034 (3.6)	26,267 (2.5)
Mental illness type	Non-SPMI	1,242,372 (81.5)	979,270 (93.0)
	SPMI	281,487 (18.5)	73,823 (7.0)
	Both	132,271 (8.7)	
Sex	Male	742,468 (48.7)	443,831 (42.2)
	Female	781,391 (51.3)	609,262 (57.9)
Age	18–24	118,327 (7.8)	
	25–34	253,850 (16.7)	
	45–54	628,150 (41.2)	
	55–64	523,532 (34.4)	
	65–74		461,664 (43.8)
	75–84		346,093 (32.9)
	85+		245,336 (23.3)
Insurance	Medicare	337,094 (22.1)	949,925 (90.2)
	Medicaid	526,836 (34.6)	14,337 (1.4)
	Private	463,685 (30.4)	67,510 (6.4)
	Self	128,089 (8.4)	4743 (0.5)
	No charge	12,392 (0.8)	307 (0.03)
	Other	55,763 (3.7)	16,271 (1.6)
Income Quartile	0–25%	537,218 (35.3)	294,736 (28.0)
	26–50%	420,002 (27.6)	284,244 (27.0)
	51–75%	333,923 (21.9)	256,369 (24.3)
	76–100%	232,716 (15.3)	217,744 (20.7)
Number of diagnoses	1–2	19,533 (1.3)	1202 (0.1)
	3–5	143,738 (9.4)	16,888 (1.6)
	6+	1,360,588 (89.3)	1,035,003 (98.3)
Comorbidity	Substance use	47,751 (3.1)	3549 (0.3)
	Alcohol disorder	275,212 (18.0)	67,321 (6.4)
	Heart failure	160,093 (10.5)	295,406 (28.0)
	Hypertension	482,459 (31.7)	440,065 (41.8)
	Chronic pain	195,882 (12.8)	116,851 (11.1)
	Diabetes	350,789 (23.0)	358,626 (34.0)
Admission type	Nonelective	1,267,817 (83.2)	903,617 (85.8)
	Elective	256,042 (16.8)	149,476 (14.2)
Hospital ownership	Government, nonfederal	191,155 (12.5)	104,359 (9.9)
	Private, not-for-profit	1,106,726 (72.6)	787,715 (74.8)
	Private, investor-owned	225,978 (14.8)	161,019 (15.3)
Bed size	Small	316,982 (20.8)	237,030 (22.5)
	Medium	432,745 (28.5)	318,591 (30.3)
	Large	774,132 (50.8)	49,7472 (47.2)
Hospital location	Urban teaching	1,094,255 (71.8)	704,517 (66.9)
	Urban nonteaching	299,377 (19.7)	238,433 (22.6)
	Rural	130,227 (8.6)	110,143 (10.5)
Patient location	Central counties of metro areas of ≥1 million population	462,933 (30.4)	290,176 (27.6)
	Fringe counties of metro areas of ≥1 million population	346,964 (22.8)	261,967 (24.9)
	Counties in metro areas of 250,000–999,999 population	324,566 (21.3)	215,766 (20.5)
	Counties in metro areas of 50,000–249,999 population	149,464 (9.8)	103,962 (9.9)
	Micropolitan counties	141,533 (9.3)	102,257 (7.5)
	Nonmetropolitan/micropolitan counties	98,399 (6.5)	78,965 (7.5)

**Table 2:** Mean LOS by Sample Characteristics (Unweighted).

		18–64	Kruskal-Wallis Test	65+	Kruskal-Wallis Test
Race	White	5.0	<0.001	5.5	<0.001
	Black	5.5		6.5	
	Hispanic	5.3		5.9	
	Asian/Pacific Islander	6.4		6.4	
	Native American/other	5.6		6.2	
Mental illness type	Non-SPMI	5.0	<0.001	5.6	<0.001
	SPMI	6.3		7.3	
Sex	Male	5.5	<0.001	6.0	<0.001
	Female	4.9		5.5	
Age	18–24	4.8	<0.001		
	25–34	4.8			
	45–54	5.1			
	55–64	5.6			
	65–74			5.7	<0.001
	75–84			5.8	
	85+			5.4	
Insurance	Medicare	5.8	<0.001	5.7	<0.001
	Medicaid	5.5		7.7	
	Private	4.7		5.7	
	Self	4.4		6.3	
	No charge	4.7		8.2	
	Other	5.1		5.6	
Income Quartile	0–25%	5.2	<0.001	5.8	<0.001
	26–50%	5.1		5.6	
	51–75%	5.1		5.6	
	76–100%	5.3		5.7	
Number of diagnoses	1–2	5.3	<0.001	6.6	<0.001
	3–5	3.9		4.1	
	6+	5.3		5.7	
Comorbidity	Substance use	5.5	<0.001	6.6	<0.001
	Alcohol disorder	5.4	<0.001	6.2	<0.001
	Heart failure	6.3	<0.001	6.3	<0.001
	Hypertension	5.1	<0.001	5.3	<0.001
	Chronic pain	5.6	<0.001	5.7	<0.001
	Diabetes	5.5	<0.001	5.9	<0.001
Admission type	Non-elective	5.3	<0.001	5.7	<0.001
	Elective	4.6		5.6	
Hospital ownership	Government, nonfederal	6.1	<0.001	6.2	<0.001
	Private, not-for-profit	5.1		5.6	
	Private, investor-owned	4.9		5.6	
Bed size	Small	4.7	<0.001	5.3	<0.001
	Medium	4.9		5.5	
	Large	5.6		6.0	
Type of hospital	Urban teaching	5.5	<0.001	5.9	<0.001
	Urban-nonteaching	4.7		5.3	
	Rural	4.3		5.1	
Patient location	Central counties of metro areas of ≥1 million population	5.5	<0.001	6.0	<0.001
	Fringe counties of metro areas of ≥1 million population	5.2		5.6	
	Counties in metro areas of 250,000–999,999 population	5.1		5.6	
	Counties in metro areas of 50,000–249,999 population	4.9		5.5	
	Micropolitan counties	4.9		5.4	
	Nonmetropolitan/micropolitan counties	4.9		5.5	

**Table 3:** Weighted Predicted Mean and Changes in Racial/Ethnic Differences in LOS between Patients with Non-SPMI vs. SPMI, by age groups.

	18–64				65+			
	Non-SPMI	SPMI	Differences in LOS between Non SPMI vs. SPMI	95% Conf. Interval	Non-SPMI	SPMI	Differences in LOS between Non SPMI vs. SPMI	95% Conf. Interval
White	4.81	6.22			5.44	7.27		
Hispanic	5.04	6.64	−0.05	(−0.17, 0.07)	6.09	7.76	−0.61 ***	(−0.88, −0.34)
Black	4.95	6.28	0.27 ***	(0.17, 0.36)	5.60	6.77	−0.08	(−0.34, 0.19)
Asian/PI	5.73	8.69	1.60 ***	(1.14, 2.06)	6.05	8.89	1.09 **	(0.19, 1.99)
Native American/other	5.22	7.37	0.76 ***	(0.54, 0.97)	5.97	8.19	0.45 ***	(0.05, 0.95)

*** *p* < 0.01, ** *p* < 0.05, * *p* < 0.1.

## Data Availability

Not applicable.
